# Supplementary material for: Personalized biomarkers of multiscale functional alterations in temporal lobe epilepsy
Source: Nat Commun. 2025 Nov 19;16:10145. doi: 10.1038/s41467-025-65042-1 (PMC12630870; doi:10.1038/s41467-025-65042-1)
Supplement: Supplementary file 1 — Supplementary Information [file 41467_2025_65042_MOESM1_ESM.pdf]

SUPPLEMENTARY INFORMATION

Personalized biomarkers of multiscale functional alterations in temporal lobe epilepsy

Ke Xie,[1*] Ella Sahlas,[1] Alexander Ngo,[1] Judy Chen,[1] Thaera Arafat,[1] Jessica Royer,[1] Yigu Zhou,[1] Raúl Rodríguez-Cruces,[1] Arielle Dascal,[1] Benoit Caldairou,[1] Fatemeh Fadaie,[1] Alexander Barnett,[1] Samantha Audrain,[1] Sara Larivière,[2] Lorenzo Caciagli,[3] Raluca Pana,[4] Alexander G. Weil,[5] Christophe Grova,[1] Birgit Frauscher,[6] Dewi V. Schrader,[7] Zhiqiang Zhang,[8] Luis Concha,[9] Andrea Bernasconi,[1#] Neda Bernasconi,[1#] and Boris C. Bernhardt[1#*]

Author Affiliations:

[1] McConnell Brain Imaging Centre, Montreal Neurological Institute and Hospital, McGill University, Montreal, QC, Canada

[2] Sherbrooke Laboratory for Integrative Connectomics, Centre de Recherche du CHUS, Université de Sherbrooke, QC, Canada

[3] Department of Neurology, Inselspital, Sleep-Wake-Epilepsy-Center, Bern University Hospital, University of Bern, Bern, Switzerland

[4] Montreal Neurological Institute and Hospital, McGill University, Montreal, QC, Canada

[5] Division of Neurosurgery, Department of Surgery, Sainte-Justine University Hospital Centre, Montreal, QC, Canada

[6] Department of Neurology, Department of Biomedical Engineering, Duke University, Durham, NC, USA

[7] British Columbia Children's Hospital, University of British Columbia, Vancouver, BC, Canada

[8] Department of Medical Imaging, Jinling Hospital, Nanjing University School of Medicine, Nanjing, China

[9] Institute of Neurobiology, Universidad Nacional Autónoma de Mexico, Queretaro, Mexico

[#] These authors jointly supervised this work

* **Correspondence to**:

Ke Xie, MSc
McConnell Brain Imaging Centre,
Montreal Neurological Institute and Hospital,
McGill University, Montreal, QC, Canada
E-Mail: ke.xie@mail.mcgill.ca

Boris C. Bernhardt, PhD
McConnell Brain Imaging Centre,
Montreal Neurological Institute and Hospital,
McGill University, Montreal, QC, Canada
E-Mail: boris.bernhardt@mcgill.ca

MRI ACQUISITION

MICA-MICs dataset. Data were collected on a 3-T Siemens Magnetom Prisma-Fit scanner equipped with a 64-channel head coil, and included: (i) two T1-weighted scans (3D-MPRAGE, repetition time [TR] = 2300 ms, echo time [TE] = 3.14 ms, flip angle [FA] = 9°, field of view [FOV] = 256×256 mm^2, voxel size = 0.8×0.8×0.8 mm^3, matrix size = 320×320, 224 slices), (ii) a resting-state functional MRI (fMRI) scan (multiband accelerated 2D-BOLD echo-planar imaging (EPI), TR = 600 ms, TE = 30 ms, FA = 52°, FOV = 240×240 mm^2, voxel size = 3×3×3 mm^3, multi-band factor = 6, 48 slices, 700 volumes), and (iii) a multi-shell diffusion MRI scan (2D spin-echo EPI, TR = 3500 ms, TE = 64.40 ms, FA = 90°, FOV = 224×224 mm^2, voxel size = 1.6×1.6×1.6 mm^3, 3 b0 images, b-values = 300/700/2000 s/mm^2 with 10/40/90 diffusion directions). During the resting-state fMRI acquisition, participants were instructed to stay still, fixate a cross presented on the screen, and not to fall asleep.

EpiC dataset. Data were collected on a 3-T Philips Achieva scanner equipped with a 64-channel head coil, and included: (i) a T1-weighted scan (3D spoiled gradient-echo, TR = 8.1 ms, TE = 3.7 ms, FA = 8°, FOV = 256×256 mm^2, voxel size = 1×1×1 mm^3, 240 slices), (ii) a resting-state fMRI scan (gradient-echo EPI, TR = 2000 ms, TE = 30 ms, FA = 90°, voxel size = 2×2×3 mm^3, 34 slices, 200 volumes), and (iii) a diffusion MRI scan (2D EPI, TR = 11.86 s, TE = 64.3 ms, FOV = 256×256 mm^2, voxel size = 2×2×2 mm^3, 2 b0 images, b-value = 2000 s/mm^2, 60 diffusion directions). During the resting-state fMRI acquisition, participants were instructed to keep their eyes closed and not to fall asleep.

Nanj dataset. Data were collected on a 3-T Siemens Trio scanner equipped with a 32-channel head coil, and included: (i) a T1-weighted scan (3D-MPRAGE, TR = 2300 ms, TE = 2.98 ms, FA = 9°, FOV = 256×256 mm^2, voxel size = 0.5×0.5×1 mm^3), (ii) a resting-state fMRI scan (2D gradient-echo EPI, TR = 2000 ms, TE = 30 ms, FA = 90°, FOV = 240×240 mm^2, voxel size = 3.75×3.75×4 mm^3, 30 slices, 255 volumes), and (iii) a diffusion MRI scan (2D spin-echo EPI, TR = 6100 ms, TE = 93 ms, FA = 90°, FOV = 240×240 mm^2, voxel size = 0.94×0.94×3 mm^3, 4 b0 images, b-value = 1000 s/mm^2, 120 diffusion directions). During the resting-state fMRI acquisition, participants were instructed to keep their eyes closed and not to fall asleep.

NOEL dataset. Data were collected on a 3-T Siemens Trio scanner equipped with a 32-channel head coil, and included: (i) a T1-weighted scan (3D-MPRAGE, TR = 2300 ms, TE = 2.98 ms, FA = 9°, voxel size = 1×1×1 mm^3), (ii) a resting-state fMRI scan (2D gradient-echo EPI, TR = 2020 ms, TE = 30 ms, FA = 90°, voxel size = 4×4×4 mm^3, 34 slices, 150 volumes), and (iii) a diffusion MRI scan (2D twice-refocused EPI, TR = 8400 ms, TE = 90 ms, FA = 90°, voxel size = 2×2×3 mm^3, 63 slices, 1 b0 images, b-value = 1000 s/mm^2, 64 diffusion directions). During the resting-state fMRI acquisition, participants were instructed to keep their eyes closed and not to fall asleep.

MRI PROCESSING

Multimodal MRI data were preprocessed using *micapipe* (v0.2.3; https://micapipe.readthedocs.io/),[1] an openly accessible multimodal MRI pipeline that integrates AFNI, FSL, FreeSurfer, ANTs, MRtrix, and Workbench.[2-6] T1-weighted MRI data were de-obliqued, reoriented to standard orientation (LPI: left to right, posterior to anterior, and inferior to superior), linearly co-registered, corrected for intensity non-uniformity, intensity normalized, skull stripped, and submitted to FreeSurfer 6.0 to extract models of the inner and outer cortical interfaces. Segmentation errors were manually corrected. Subject-specific cortical thickness was measured as Euclidean distance between corresponding pial and white matter vertices, and mapped to the hemisphere-matched fsLR-32k surface template (~32k vertices/hemisphere) using workbench tools.[7-9]

Diffusion MRI data were denoised, and corrected for susceptibility distortions, head motion, and eddy currents using MRtrix3 (http://www.mrtrix.org/).[6] A Laplacian potential field was used to guide the placement of a superior white matter (SWM) surface, targeting a depth of ~2 mm beneath the gray-white matter boundary.[10,11] Diffusion features, fractional anisotropy (FA) and mean diffusivity (MD), which serve as surrogates of fiber architecture and tissue microstructure, were linearly interpolated along the SWM surface and mapped to the fsLR-32k surface template.

Resting-state fMRI preprocessing included discarding the first five volumes, reorientation, slice-timing correction, correction for head motion and distortion, and temporal band-pass filtering (0.01–0.08 Hz). Motion correction was performed using a rigid-body model with 6 parameters by registering all timepoint volumes to the mean volume. Motion outlier volumes (spikes) were discarded using FSL's motion outlier detection outputs.[1] To maintain the temporal continuity of timeseries, we subsequently filled these censored frames using a linear interpolation. Nuisance signals removal was conducted either using an in-house trained ICA-FIX classifier (for *MICs*),[12] or through regression of white matter and cerebrospinal fluid signals for other datasets. FreeSurfer surfaces were non-linearly registered to the native average volumetric timeseries using label-based registration. Cortical timeseries were then mapped to the fsLR-32k surface template from the native fMRI space using trilinear interpolation. Subject-specific subcortical parcellations were non-linearly registered to each individual's native fMRI space using the deformable SyN approach implemented in ANTs.[5]

Surface-based maps, including cortical thickness, FA, MD, and resting-state fMRI time series, were smoothed along the cortical sheet with a 10-mm FWHM Gaussian kernel. This surface-constrained smoothing improves signal-to-noise ratio, decreases variability, and retains sensitivity while limiting partial-volume blurring across tissue boundaries.[1,9,13,14] Subsequently, vertex-wise cortical maps were parcellated using the HCPMMP1.0 atlas (or Glasser atlas),[15] a multimodal cortical parcellation comprising 180 homologous regions per hemisphere.

REFERENCES

1. Rodriguez-Cruces, R. et al. Micapipe: a pipeline for multimodal neuroimaging and connectome analysis. *NeuroImage* **263**, 119612 (2022).
2. Cox, R. W. AFNI: Software for analysis and visualization of functional magnetic resonance neuroimages. *Comput. Biomed. Res.* **29**, 162-173 (1996).
3. Jenkinson, M., Beckmann, C. F., Behrens, T. E. J., Woolrich, M. W. & Smith, S. M. FSL. *NeuroImage* **62**, 782-790 (2012).
4. Fischl, B. FreeSurfer. *NeuroImage* **62**, 774-781 (2012).
5. Avants, B. B., Epstein, C. L., Grossman, M. & Gee, J. C. Symmetric diffeomorphic image registration with cross-correlation: Evaluating automated labeling of elderly and neurodegenerative brain. *Med. Image Anal.* **12**, 26-41 (2008).
6. Tournier, J. D. et al. MRtrix3: A fast, flexible and open software framework for medical image processing and visualisation. *NeuroImage* **202**, 116137 (2019).
7. Van Essen, D. C., Glasser, M. F., Dierker, D. L., Harwell, J. & Coalson, T. Parcellations and hemispheric asymmetries of human cerebral cortex analyzed on surface-based atlases. *Cereb. Cortex* **22**, 2241-2262 (2012).
8. Marcus, D. S. et al. Human connectome project informatics: quality control, database services, and data visualization. *NeuroImage* **80**, 202-219 (2013).

9. Glasser, M. F. et al. The minimal preprocessing pipelines for the Human Connectome Project. *NeuroImage* **80**, 105-124 (2013).
10. Liu, M. et al. The superficial white matter in temporal lobe epilepsy: a key link between structural and functional network disruptions. *Brain* **139**, 2431-2440 (2016).
11. Larivière, S. et al. Functional connectome contractions in temporal lobe epilepsy: Microstructural underpinnings and predictors of surgical outcome. *Epilepsia* **61**, 1221-1233 (2020).
12. Salimi-Khorshidi, G. et al. Automatic denoising of functional MRI data: Combining independent component analysis and hierarchical fusion of classifiers. *NeuroImage* **90**, 449-468 (2014).
13. Royer, J. et al. An open MRI dataset for multiscale neuroscience. *Sci. Data* **9**, 569 (2022).
14. Lerch, J. P. & Evans, A. C. Cortical thickness analysis examined through power analysis and a population simulation. *NeuroImage* **24**, 163-173 (2005).
15. Glasser, M. F. et al. A multi-modal parcellation of human cerebral cortex. *Nature* **536**, 171-178 (2016).

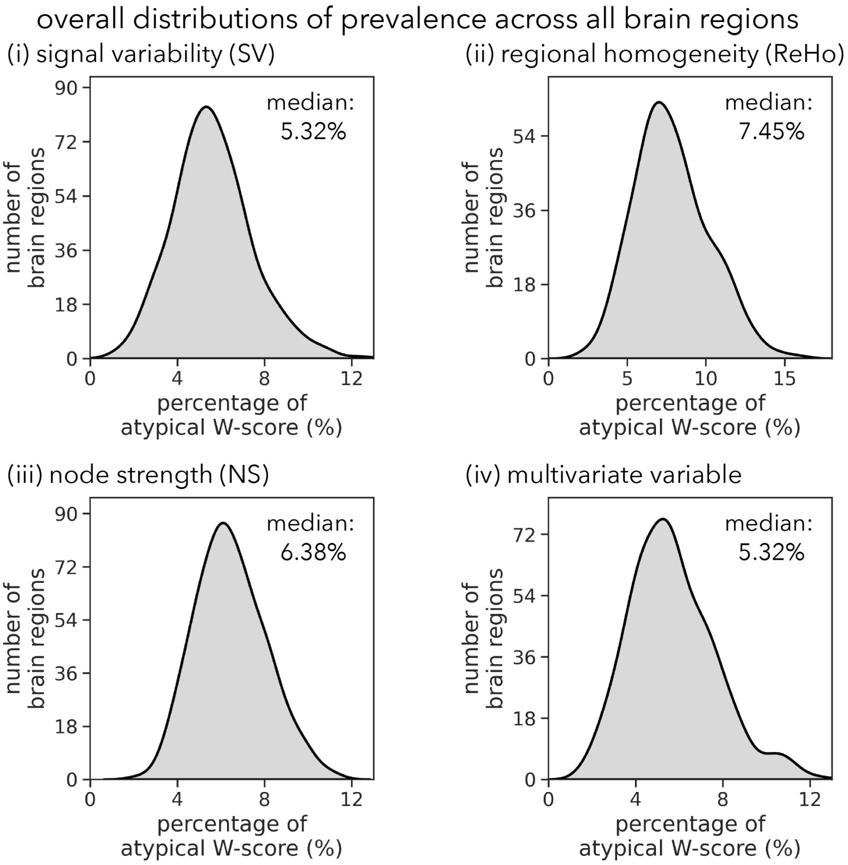

Fig. S1 | Distributions of the regional deviation prevalence. Histograms showing the across-region distributions of the proportion of TLE patients with extreme deviations ($|W\text{-score}| \geq 1.96$) for (***i***) signal variability, (***ii***) regional homogeneity, (***iii***) node strength, and (***iv***) a multivariate composite. Source data are provided as a Source Data file.

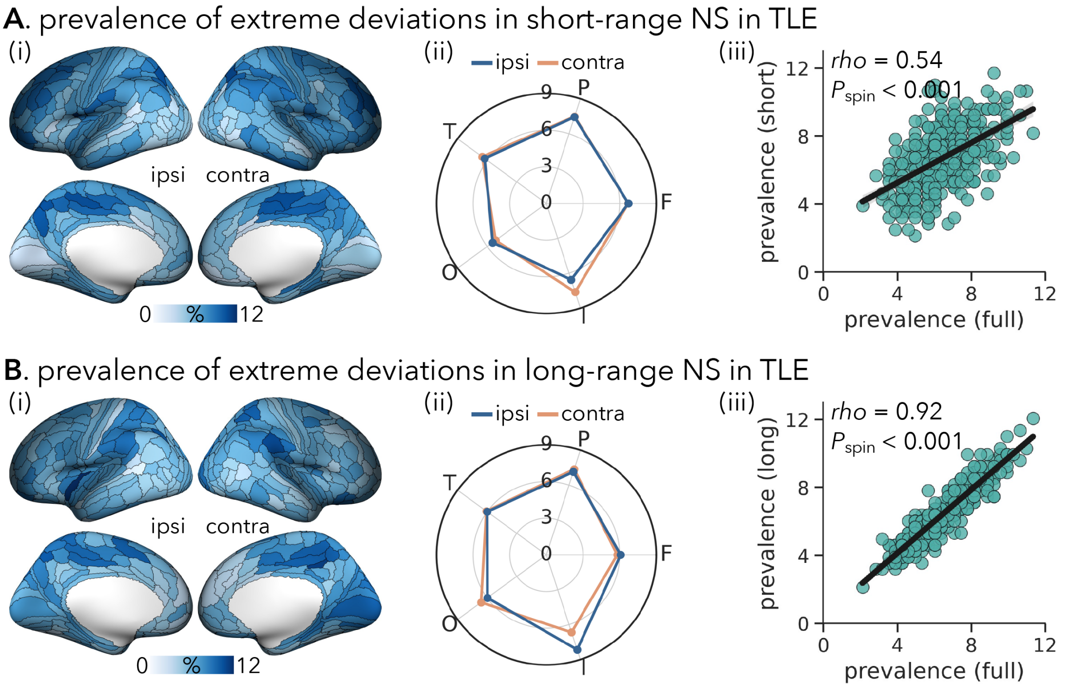

Fig. S2 | Region-specific prevalence of extreme deviations in TLE patients in short-range (A) and long-range NS (B). (***i***) Proportion of patients with extreme deviations (|*W*-score| ≥ 1.96) in each region. (***ii***) Mean proportion of extreme deviations in each lobe. (***iii***) Spatial correlations between full-range NS deviations (x-axis; from **Fig. 2A**) and short-range and long-range NS deviations (y-axis); dots denote brain regions. Statistical significance (*i.e.*, P_{spin}) of observed Spearman correlations is assessed using spin permutation tests with 5,000 iterations (one-sided). Abbreviation: NS = node strength; ipsi = ipsilateral; contra = contralateral; F = frontal; P = parietal; T = temporal; O = occipital; I = insula. Source data are provided as a Source Data file.

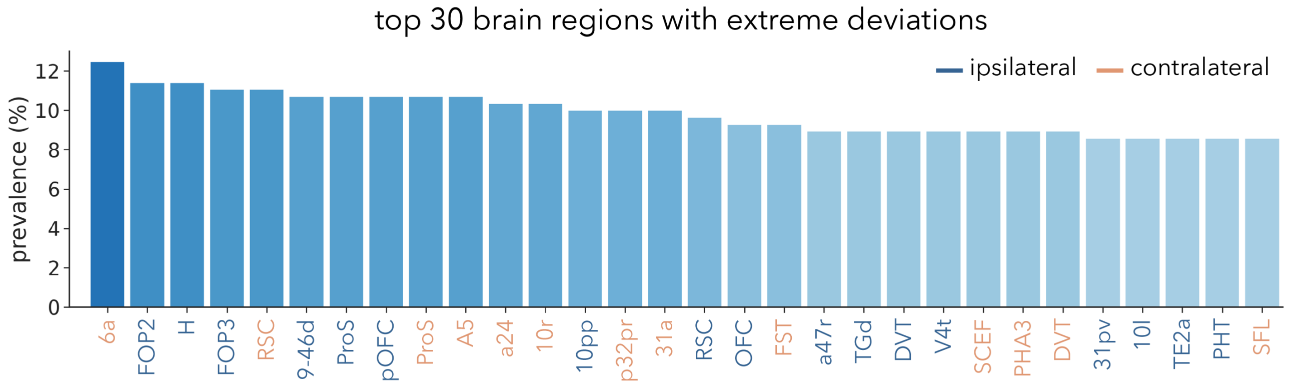

Fig. S3 | Top regions by prevalence of extreme functional deviations. The bar chart lists the 30 brain regions with the highest proportion of patients showing extreme functional deviations ($|W\text{-score}| \geq 1.96$) in TLE, colored by the prevalence values (blue = ipsilateral; orange = contralateral). Source data are provided as a Source Data file.

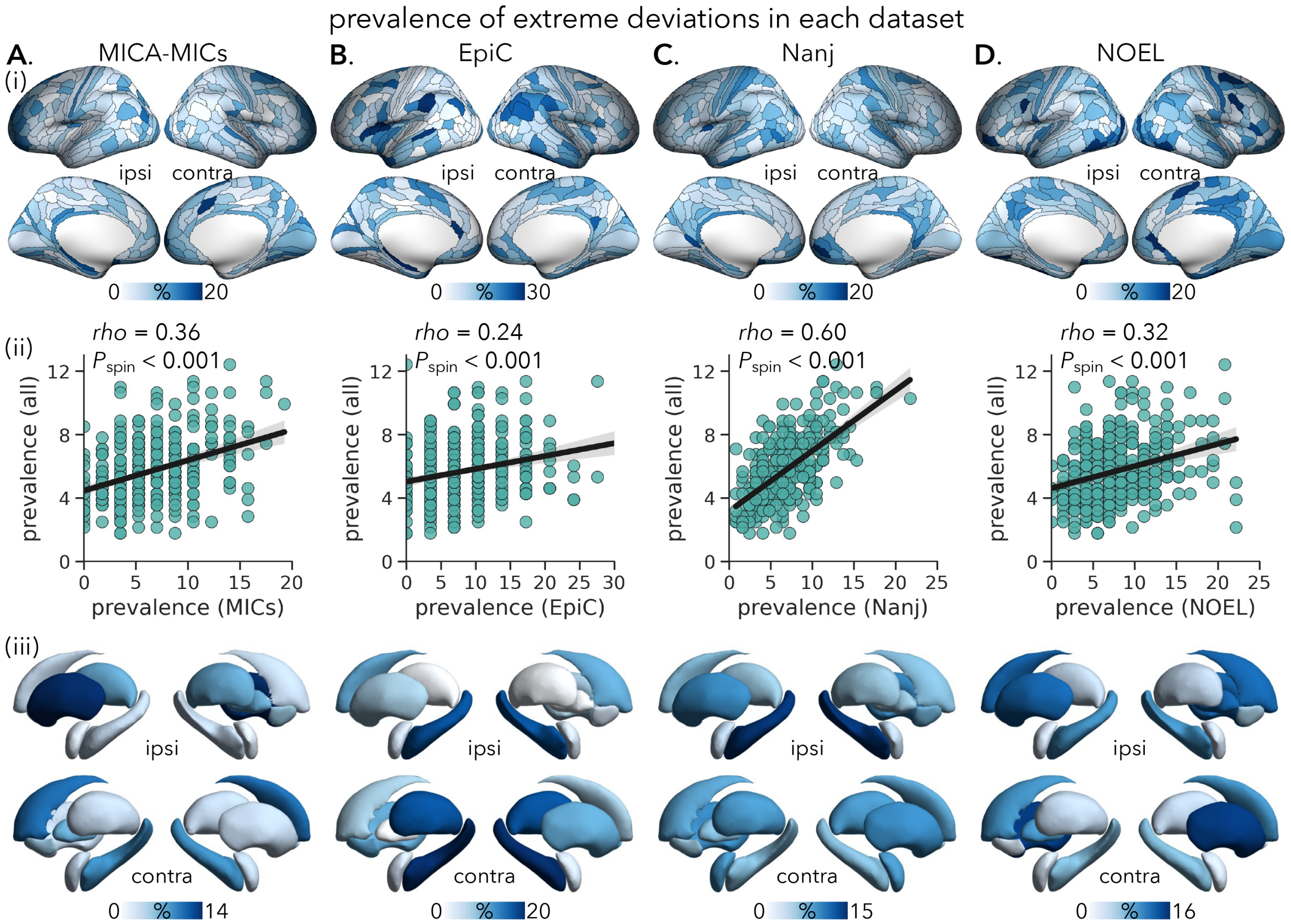

Fig. S4 | Region-specific prevalence of extreme functional deviations in TLE by dataset. (***i***): Proportion of patients with extreme deviations (|*W*-score| ≥ 1.96) per region shown separately in (**A**) MICA-MICs, (**B**) EpiC, (**C**) Nanj, and (**D**) NOEL. (***ii***): Spatial correlations between dataset-specific regional deviation prevalence map (x-axis) and the four-dataset pooled map (y-axis; from **Fig. 2B (*i*)**); dots denote brain regions. Error band (shadows in gray) denotes the 95% confidence interval around the fit. Statistical significance (*i.e.*, P_{spin}) of Spearman correlation is assessed using spin permutation tests (5,000 iterations; one-sided). (***iii***) Proportion of patients with extreme deviations in subcortical structures for each dataset. Abbreviation: ipsi = ipsilateral; contra = contralateral. Source data are provided as a Source Data file.

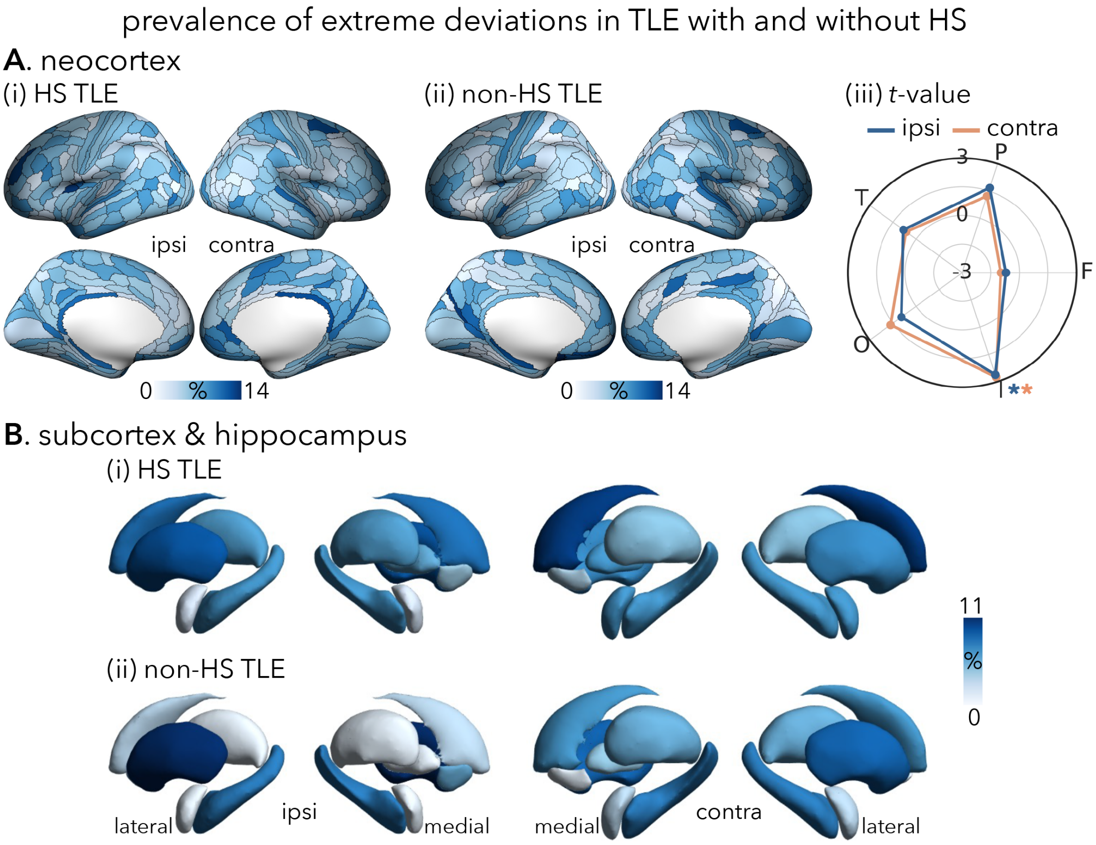

Fig. S5 | Region-specific prevalence of extreme functional deviations in TLE patients with and without hippocampal sclerosis (HS). (**A**) Extreme deviations in neocortex. Proportion of patients with extreme deviations (|*W*-score| ≥ 1.96) in each brain region in (***i***) HS TLE (n = 192) and (***ii***) non-HS TLE (n = 90) subgroups separately. (***iii***) Differences (paired *t*-test) in the number of extreme deviations between HS and non-HS patients in each lobe. (**B**) Extreme deviations in subcortex and hippocampus. Proportion of patients with extreme deviations in each subcortical structure and the hippocampus in (***i***) HS and (***ii***) non-HS TLE subgroups separately. * $P_{FDR} < 0.05$. Abbreviation: ipsi = ipsilateral; contra = contralateral; F = frontal; P = parietal; T = temporal; O = occipital; I = insula. Source data are provided as a Source Data file.

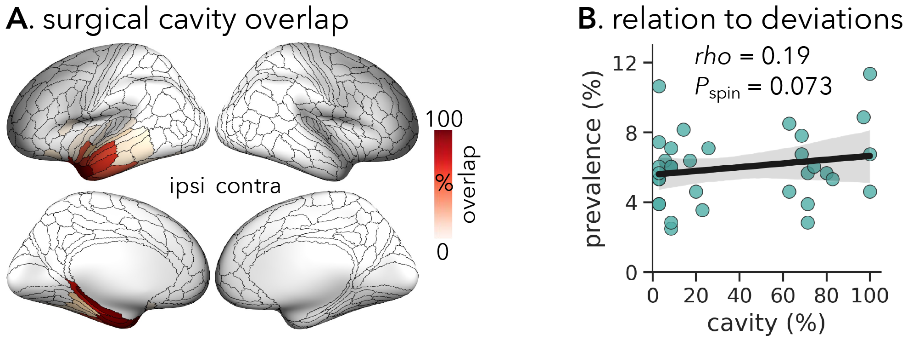

Fig. S6 | Spatial correlation between the overlap of surgical cavities and TLE-related functional deviations. (**A**) The overlap map of surgical cavities segmented from pre- and post-surgical T1-weighted MRIs across 35 TLE patients. (**B**) Spearman correlation between the overlap map of surgical cavities and the proportion of extreme deviations within the resected brain regions after spin permutation tests (5,000 iterations; one-sided). Error band (shadows in gray) denotes the 95% confidence interval around the fit. Abbreviation: ipsi = ipsilateral; contra = contralateral. Source data are provided as a Source Data file.

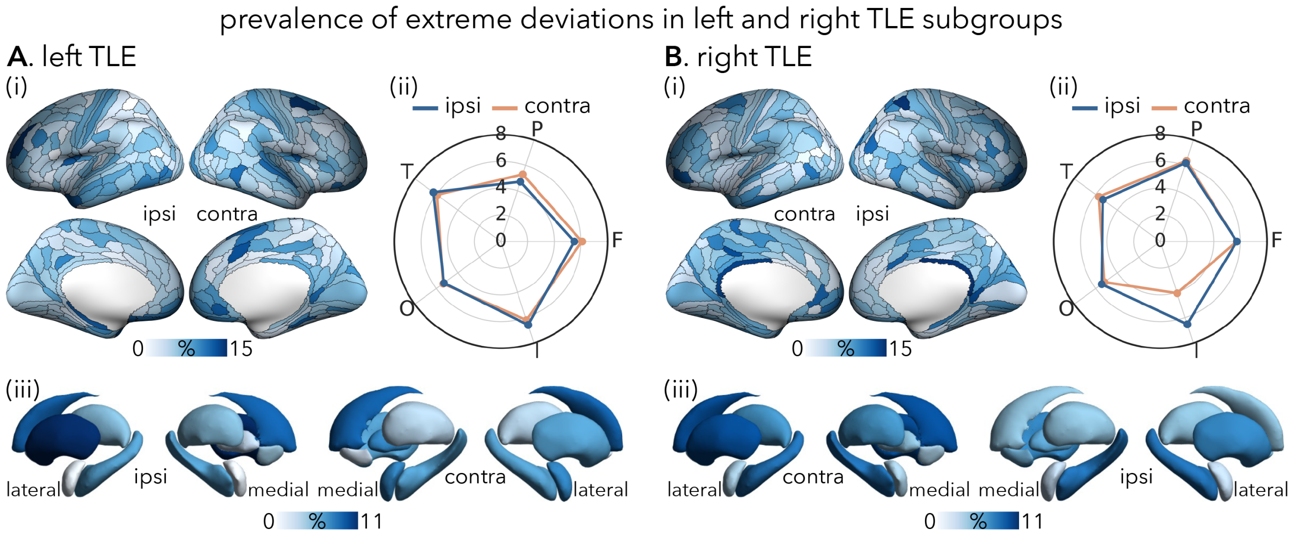

Fig. S7 | Region-specific prevalence of extreme functional deviations in (A) left and (B) right TLE subgroups. Proportion of patients with extreme deviations (|W-score| ≥ 1.96) in each (***i***) brain region, (***ii***) lobe, and (***iii***) subcortical structure in left TLE (n = 147) and right TLE (n = 135) subgroups. Abbreviation: ipsi = ipsilateral; contra = contralateral; F = frontal; P = parietal; T = temporal; O = occipital; I = insula. Source data are provided as a Source Data file.

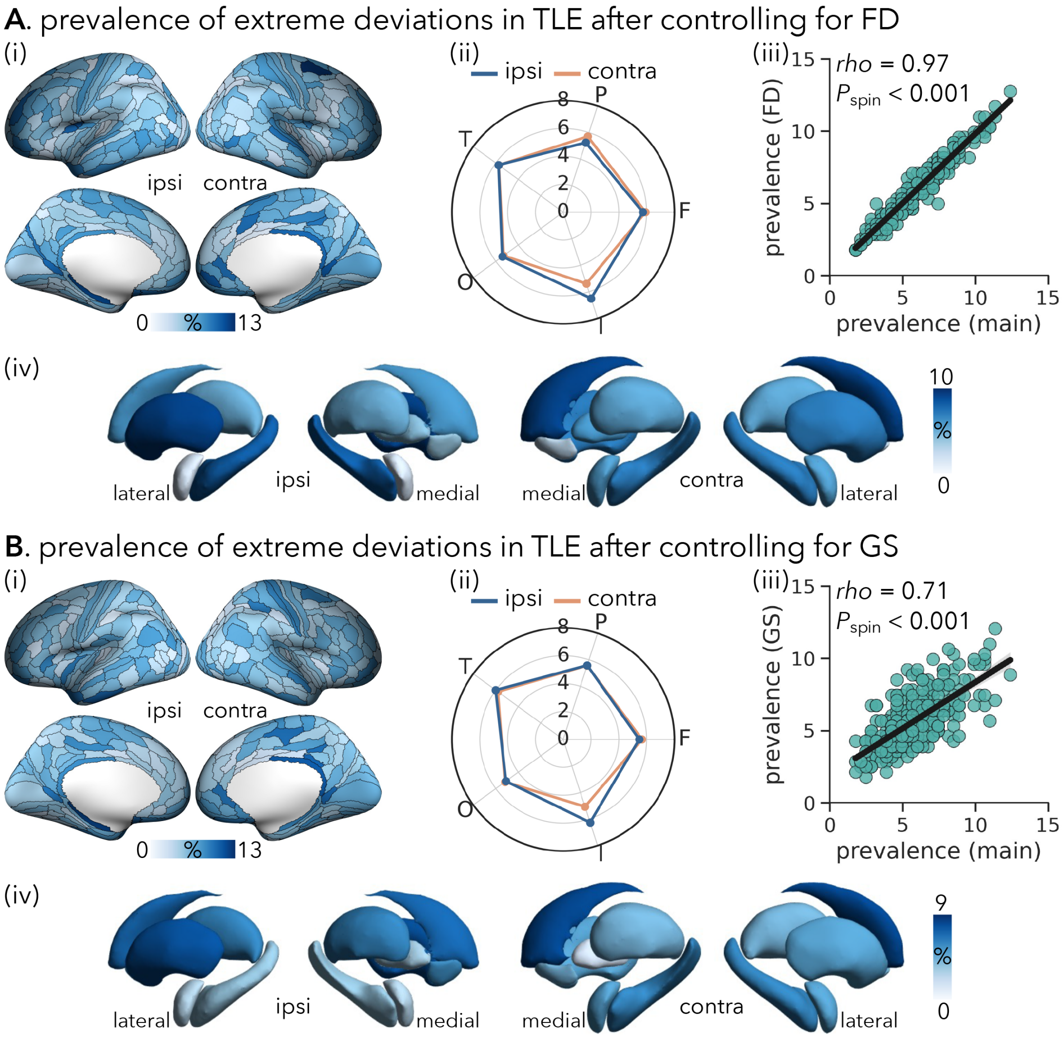

Fig. S8 | Region-specific prevalence of extreme functional deviations in TLE patients after additionally controlling for (A) head motion or (B) global signal. (***i***) Proportion of patients with extreme deviations (|*W*-score| ≥ 1.96) in each cortical region when regressing out head motion (measured by framewise displacement (FD)) in rs-fMRI scans or global mean signal (GS). (***ii***) Mean proportion of extreme deviations in each lobe. (***iii***) Spatial correlations between the deviation prevalence patterns before (x-axis; from **Fig. 2B** (***i***)) and after (y-axis) controlling for FD or GS; dots denote brain regions. Error band (shadows in gray) denotes the 95% confidence interval around the fit. (***iv***) Proportions of patients with extreme deviations in subcortical structures and the hippocampus when controlling for FD or GS. Abbreviation: ipsi = ipsilateral; contra = contralateral; F = frontal; P = parietal; T = temporal; O = occipital; I = insula. Source data are provided as a Source Data file.
