# Supplementary material for: Personalized biomarkers of multiscale functional alterations in temporal lobe epilepsy
Source: Nat Commun. 2025 Nov 19;16:10145. doi: 10.1038/s41467-025-65042-1 (PMC12630870; doi:10.1038/s41467-025-65042-1)
Supplement: Supplementary file 2 — Reporting Summary [file 41467_2025_65042_MOESM2_ESM.pdf]

nature portfolio

Corresponding author(s): Boris Bernhardt

Last updated by author(s): Sep 26, 2025

Reporting Summary

Nature Portfolio wishes to improve the reproducibility of the work that we publish. This form provides structure for consistency and transparency in reporting. For further information on Nature Portfolio policies, see our Editorial Policies and the Editorial Policy Checklist.

Statistics

For all statistical analyses, confirm that the following items are present in the figure legend, table legend, main text, or Methods section.

n/a	Confirmed	
☐	☒	The exact sample size (*n*) for each experimental group/condition, given as a discrete number and unit of measurement
☐	☒	A statement on whether measurements were taken from distinct samples or whether the same sample was measured repeatedly
☐	☒	The statistical test(s) used AND whether they are one- or two-sided<br>*Only common tests should be described solely by name; describe more complex techniques in the Methods section.*
☐	☒	A description of all covariates tested
☐	☒	A description of any assumptions or corrections, such as tests of normality and adjustment for multiple comparisons
☐	☒	A full description of the statistical parameters including central tendency (e.g. means) or other basic estimates (e.g. regression coefficient) AND variation (e.g. standard deviation) or associated estimates of uncertainty (e.g. confidence intervals)
☐	☒	For null hypothesis testing, the test statistic (e.g. *F*, *t*, *r*) with confidence intervals, effect sizes, degrees of freedom and *P* value noted<br>*Give P values as exact values whenever suitable.*
☒	☐	For Bayesian analysis, information on the choice of priors and Markov chain Monte Carlo settings
☒	☐	For hierarchical and complex designs, identification of the appropriate level for tests and full reporting of outcomes
☐	☒	Estimates of effect sizes (e.g. Cohen's *d*, Pearson's *r*), indicating how they were calculated

Our web collection on statistics for biologists contains articles on many of the points above.

Software and code

Policy information about availability of computer code

Data collection	Proprietary software for the 3.0-Tesla MRI scanners (Siemens, Philips) were used to collect the neuroimaging data.
Data analysis	MRI preprocessing was conducted using micapipe (v0.2.3; http://micapipe.readthedocs.io). Code for spin permutation testing is available at https://github.com/frantisekvasa/rotate_parcellation. Code for dominance analysis is available at https://netneurolab.github.io/netneurotools. LIBSVM toolbox used for supervised machine learning is available at https://github.com/cjlin1/libsvm (v3.31). The visualiza-tion of brain mapping images was conducted using the ENIGMA Toolbox (v2.0.1; https://enigma-toolbox.readthedocs.io). Costume scripts used for analysis are available at https://github.com/MICA-MNI/TLE_rsfmri-deviations.

For manuscripts utilizing custom algorithms or software that are central to the research but not yet described in published literature, software must be made available to editors and reviewers. We strongly encourage code deposition in a community repository (e.g. GitHub). See the Nature Portfolio guidelines for submitting code & software for further information.

Data

Policy information about availability of data

All manuscripts must include a data availability statement. This statement should provide the following information, where applicable:

- Accession codes, unique identifiers, or web links for publicly available datasets
- A description of any restrictions on data availability
- For clinical datasets or third party data, please ensure that the statement adheres to our policy

Raw data from the MICA-MICs dataset are available via the Canadian Open Neuroscience Platform (CONP: https://portal.conp.ca/) and the Open Science Framework (OSF: https://osf.io/j532r/). Raw data from the EpiC dataset are available on OpenNeuro (data set ds004469, https://openneuro.org/datasets/ds004469/versions/1.1.4). Access to raw data from the Nanj and NO-EL datasets can be granted upon reasonable request, subject to institutional review and the signing of a data use agreement outlining the terms of access, use, storage and authorship. Source data are provid-ed with this paper.

Research involving human participants, their data, or biological material

Policy information about studies with human participants or human data. See also policy information about sex, gender (identity/presentation), and sexual orientation and race, ethnicity and racism.

Reporting on sex and gender	We included both male and female participants, with sex determined by self-report. The sex composition of the dataset is reported in the main text. Additional effects of gender identify were not specifically assessed.
Reporting on race, ethnicity, or other socially relevant groupings	There were no socially constructed nor socially relevant categorization variables used in this manuscript.
Population characteristics	This study included 625 participants: 298 healthy controls (152 males; mean ± SD age = 29.3 ± 8.0 years, range: 18–60 years), 282 patients with unilateral temporal lobe epilepsy (TLE) (133 males; 32.3 ± 10.0 years [18–64 years]), 45 disease controls with extratemporal focal cortical dysplasia (FCD) (23 males; 27.2 ± 7.9 years [18–54 years]). 75.9% of TLE patients (n = 214) and 88.9% of FCD patients (n = 40) had drug-resistant seizures. For TLE patients, the mean ± SD age at seizure onset was 17.8 ± 10.7 years [0.3–60 years] with a mean ± SD disease duration of 14.4 ± 11.0 years [0.8–49 years]. For FCD patients, the mean ± SD age at seizure onset was 11.5 ± 6.9 years [0.3–27 years], with a mean ± SD disease duration of 15.9 ± 10.3 years [0.5–48 years]. 68.1% of TLE patients (n = 192) had ipsilateral hippocampal sclerosis (HS) (Figure 1A), 6.4% (n = 18) had ipsilateral hippocampal gliosis; and 25.5% (n =72) were MRI-negative. Among the 99 TLE patients who underwent temporal lobe resections, 74 (75%) achieved seizure freedom (Engel class I) and 25 (25%) continued to experience seizures (Engel class II–IV) at a mean follow-up period of 44 ± 34 months.
Recruitment	Patients and controls were recruited from three different study sites.
Ethics oversight	The Institutional Ethics Committee at each institution approved the study procedures (MICA-MICs and NOEL datasets: Montreal Neurological Institute-Hospital, McGill University; EpiC dataset: Institute of Neurobiology, Universidad Nacional Autónoma de Mexico; Nanj dataset: Jinling Hospital, Nanjing University School of Medicine). All participants provided written informed consent in accordance with the Declaration of Helsinki.

Note that full information on the approval of the study protocol must also be provided in the manuscript.

Field-specific reporting

Please select the one below that is the best fit for your research. If you are not sure, read the appropriate sections before making your selection.

☒ Life sciences ☐ Behavioural & social sciences ☐ Ecological, evolutionary & environmental sciences

For a reference copy of the document with all sections, see nature.com/documents/nr-reporting-summary-flat.pdf

Life sciences study design

All studies must disclose on these points even when the disclosure is negative.

Sample size	This study included 625 participants: 298 healthy controls, 282 TLE patients, and 45 disease controls with FCD. Data were aggregated from four independent datasets across three epilepsy centers: (i) Montreal Neurological Institute-Hospital (MICA-MICs dataset: 100/57/17; NOEL dataset: 42/72/28), (ii) Universidad Nacional Autónoma de México (EpiC dataset: 34/29), and (iii) Jinling Hospital (Nanj dataset: 122/124). Sample size was determined based on feasibility considerations.
Data exclusions	We excluded patients with mass lesions (e.g., tumors, vascular malformations, or malformations of cortical development), a history of traumatic brain injury or encephalitis, and/or comorbid psychiatric disorders.
Replication	We repeated the main analyses independently in each dataset and observed results consistent with the pooled multicenter findings.
Randomization	N/A

Blinding | N/A

Reporting for specific materials, systems and methods

We require information from authors about some types of materials, experimental systems and methods used in many studies. Here, indicate whether each material, system or method listed is relevant to your study. If you are not sure if a list item applies to your research, read the appropriate section before selecting a response.

Materials & experimental systems

n/a	Involved in the study
☒	☐ Antibodies
☒	☐ Eukaryotic cell lines
☒	☐ Palaeontology and archaeology
☒	☐ Animals and other organisms
☒	☐ Clinical data
☒	☐ Dual use research of concern
☒	☐ Plants

Methods

n/a	Involved in the study
☒	☐ ChIP-seq
☒	☐ Flow cytometry
☐	☒ MRI-based neuroimaging

Plants

Seed stocks	N/A
Novel plant genotypes	N/A
Authentication	N/A

Magnetic resonance imaging

Experimental design

Design type	Resting state
Design specifications	N/A
Behavioral performance measures	N/A

Acquisition

Imaging type(s)	Structural, functional and diffusion MRI scans were collected in this study.
Field strength	3.0 Tesla
Sequence & imaging parameters	MICA-MICs dataset. Structural MRI: 3D-MPRAGE, TR = 2300 ms, TE = 3.14 ms, FA = 9°, FOV = 256×256 mm2, voxel size = 0.8×0.8×0.8 mm3, matrix size = 320×320, 224 slices. Resting-state functional MRI: multiband accelerated 2D-BOLD EPI, TR = 600 ms, TE = 30 ms, FA = 52°, FOV = 240×240 mm2, voxel size = 3×3×3 mm3, multi-band factor = 6, 48 slices, 700 volumes. Diffusion MRI: multi-shell, 2D spin-echo EPI, TR = 3500 ms, TE = 64.40 ms, FA = 90°, FOV = 224×224 mm2, voxel size = 1.6×1.6×1.6 mm3, 3 b0 images, b-values = 300/700/2000 s/mm2 with 10/40/90 diffusion directions.<br><br>EpiC dataset. Structural MRI: 3D spoiled gradient-echo, TR = 8.1 ms, TE = 3.7 ms, FA = 8°, FOV = 256×256 mm2, voxel size = 1×1×1 mm3, 240 slices. Resting-state functional MRI: gradient-echo EPI, TR = 2000 ms, TE = 30 ms, FA = 90°, voxel size = 2×2×3 mm3, 34 slices, 200 volumes. Diffusion MRI: 2D EPI, TR = 11.86 s, TE = 64.3 ms, FOV = 256×256 mm2, voxel size = 2×2×2 mm3, 2 b0 images, b-value = 2000 s/mm2, 60 diffusion directions.<br><br>Nanj dataset. Structural MRI: 3D-MPRAGE, TR = 2300 ms, TE = 2.98 ms, FA = 9°, FOV = 256×256 mm2, voxel size = 0.5×0.5×1 mm3. Resting-state functional MRI: 2D gradient-echo EPI, TR = 2000 ms, TE = 30 ms, FA = 90°, FOV = 240×240 mm2, voxel size = 3.75×3.75×4 mm3, 30 slices, 255 volumes. Diffusion MRI: 2D spin-echo EPI, TR = 6100 ms, TE = 93 ms, FA = 90°, FOV = 240×240 mm2, voxel size = 0.94×0.94×3 mm3, 4 b0 images, b-value = 1000 s/mm2, 120 diffusion directions.

NOEL dataset. Structural MRI: 3D-MPRAGE, TR = 2300 ms, TE = 2.98 ms, FA = 9°, voxel size = 1×1×1 mm3. Resting-state functional MRI: 2D gradient-echo EPI, TR = 2020 ms, TE = 30 ms, FA = 90°, voxel size = 4×4×4 mm3, 34 slices, 150 volumes. Diffusion MRI: 2D twice-refocused EPI, TR = 8400 ms, TE = 90 ms, FA = 90°, voxel size = 2×2×3 mm3, 63 slices, 1 b0 images, b-value = 1000 s/mm2, 64 diffusion directions.

Area of acquisition

A whole brain scan was used.

Diffusion MRI ☒ Used ☐ Not used

Parameters

MICA-MICs dataset: multi-shell, 2D spin-echo EPI, TR = 3500 ms, TE = 64.40 ms, FA = 90°, FOV = 224×224 mm2, voxel size = 1.6×1.6×1.6 mm3, 3 b0 images, b-values = 300/700/2000 s/mm2 with 10/40/90 diffusion directions.

EpiC dataset: 2D EPI, TR = 11.86 s, TE = 64.3 ms, FOV = 256×256 mm2, voxel size = 2×2×2 mm3, 2 b0 images, b-value = 2000 s/mm2, 60 diffusion directions.

Nanj dataset: 2D spin-echo EPI, TR = 6100 ms, TE = 93 ms, FA = 90°, FOV = 240×240 mm2, voxel size = 0.94×0.94×3 mm3, 4 b0 images, b-value = 1000 s/mm2, 120 diffusion directions.

NOEL dataset: 2D twice-refocused EPI, TR = 8400 ms, TE = 90 ms, FA = 90°, voxel size = 2×2×3 mm3, 63 slices, 1 b0 images, b-value = 1000 s/mm2, 64 diffusion directions.

Preprocessing

Preprocessing software

MRI data were preprocessed using micapipe (v0.2.3; https://micapipe.readthedocs.io/), an openly accessible multimodal MRI pipeline that integrates AFNI, FSL, FreeSurfer, ANTs, MRtrix, and Workbench. Structural MRI data were de-obliqued, reoriented to standard orientation, linearly co-registered, corrected for intensity non-uniformity, intensity normalized, skull stripped, and submitted to FreeSurfer 6.0 to extract models of the inner and outer cortical interfaces. Subject-specific cortical thickness was measured as Euclidean distance between corresponding pial and white matter vertices, and registered to the Conte69 template surface (~32k vertices/hemisphere). Resting-state functional MRI preprocessing included discarding the first five volumes, reorientation, slice-timing correction, and head motion correction. Nuisance signals were removed using an in-house trained ICA-FIX classifier. Preprocessed time series were non-linearly registered to native FreeSurfer space using boundary-based registration and mapped to the Conte69 template surface. Diffusion MRI data were denoised, and corrected for susceptibility distortions, head motion, and eddy currents using MRtrix3. Diffusion features, fractional anisotropy and mean diffusivity, were linearly interpolated along the SWM surface and registered to the Conte69 template surface. Surface-based maps, including cortical thickness, time series, fractional anisotropy and mean diffusivity, were smoothed using a 10-mm FWHM Gaussian kernel.

Normalization

Surface registration

Normalization template

fsaverage and Conte69

Noise and artifact removal

Structural MRI data were reoriented, linearly co-registered, corrected for intensity nonuniformity, and intensity normalized. As for resting-state functional MRI data, we applied FMRIB's ICA-based X-noiseifier (ICA-FIX) and spike regression to remove timepoints with large motion spikes, effectively removing nuisance signals. Diffusion MRI data underwent denoising b0 intensity normalization, and correction for susceptibility distortion, head motion, and eddy currents.

Volume censoring

Yes, but no scrubbing

Statistical modeling & inference

Model type and settings

Univariate, multivariate, predictive analysis; fixed effects

Effect(s) tested

Effect of task was not estimated in this study; ANOVA is not used.

Specify type of analysis: ☒ Whole brain ☐ ROI-based ☐ Both

Statistic type for inference

(See Eklund et al. 2016)

Vertex-wise timeseries, cortical thickness, fractional anisotropy and mean diffusivity data were generated from each individual. The Glasser-360 atlas with 360 brain regions across the entire cortex was used in this study.

Correction

False Discovery Rate (FDR) corrections were applied to correct for multiple comparisons. As for spatial associations, p-values were corrected for spatial autocorrelations using 5,000 spin permutation tests.

Models & analysis

n/a	Involved in the study
☐	☒ Functional and/or effective connectivity
☒	☐ Graph analysis
☐	☒ Multivariate modeling or predictive analysis

Functional and/or effective connectivity	Pearson correlation coefficients between vertex-wise time series were calculated to generate the functional connectivity.
Multivariate modeling and predictive analysis	Multilinear regression model was used to assess the associations between structural and functional metrics across the cortex. Dominance analysis was used to determine the relative contributions of structural metrics to the prediction of brain function metrics.
